# Redefining Procedural Success After Transcatheter Aortic Valve Implantation: Why Nonfatal Complications Matter

**DOI:** 10.1016/j.shj.2026.101102

**Published:** 2026-07-22

**Authors:** Marisa Avvedimento

**Affiliations:** a Quebec Heart & Lung Institute, Laval University, Quebec City, Canada; b Barcelona Clinical Coordinating Center, Mon Clínic Foundation, Barcelona, Spain

Transcatheter aortic valve implantation (TAVI) has undergone a remarkable evolution over the past 2 decades in becoming an established therapy across the entire spectrum of surgical risk.^1^ Continuous improvements in device technology, procedural planning, and operator experience have translated into a substantial reduction in periprocedural complications and marked improvements in clinical outcomes. However, as TAVI expands toward younger populations with longer life expectancy, the concept of procedural success is evolving. Achieving optimal valve implantation and early survival is no longer sufficient; contemporary TAVI programs must also provide durable clinical benefit while preserving functional status and quality of life over time.^2^^,^^3^ In this context, complications traditionally considered “nonfatal” should not be viewed as transient procedural events, as they may substantially influence the subsequent trajectory of recovery and long-term outcomes.

In this issue of *Structural Heart*, Tobe et al.^4^ report the results of a post-hoc analysis of the randomized LANDMARK trial, providing important insights into the prognostic relevance of early nonfatal complications. The authors evaluated 742 patients who survived the first 30 days after the procedure to determine whether early adverse events influenced subsequent mortality and quality of life. Complications were defined according to the Valve Academic Research Consortium–3 (VARC-3) early safety composite endpoint, including stroke, type 3 bleeding, major vascular complications, acute kidney injury, moderate or severe prosthetic valve regurgitation, new permanent pacemaker implantation, and device- or procedure-related reintervention. Overall, 177 patients (23.9%) experienced at least 1 early nonfatal event. Despite similar baseline characteristics and surgical risk profiles, patients with an early complication had a significantly higher risk of mortality between 30 days and 1 year than those with an uncomplicated postprocedural course (8.0% vs. 3.9%). After adjustment for potential confounders, the occurrence of any early adverse event remained independently associated with an approximately twofold increase in mortality risk (hazard ratio: 2.14; 95% CI: 1.07–4.27). Importantly, the prognostic impact was not uniform across the individual components of the composite endpoint. Moderate or severe prosthetic valve regurgitation, acute kidney injury, and major vascular complications were each associated with a substantially increased risk of mortality, whereas new permanent pacemaker implantation was not. Among patients surviving to 1 year, quality of life assessed by the Short Form-12 (SF-12) questionnaire was comparable between groups.

The introduction of the VARC-3 early safety endpoint at 30 days marked an important evolution in the evaluation of TAVI outcomes. By integrating mortality with major procedure- and device-related complications, VARC-3 broadened the definition of procedural success beyond technical performance and early survival.^5^ Subsequent studies confirmed its clinical relevance in demonstrating that approximately one-third of contemporary patients fail to achieve early safety and experience a significantly higher risk of mortality.^6^ However, it remained unclear whether this association simply reflected the prognostic impact of fatal periprocedural events or whether nonfatal complications themselves carried persistent prognostic implications. By restricting the analysis to patients surviving the first 30 days, Tobe et al. address this gap and demonstrate that nonfatal complications carry prognostic information independent of early mortality.

Nevertheless, these findings should be interpreted in the context of the heterogeneous nature of the study endpoint, as its individual components differ substantially in terms of mechanisms, severity, and clinical consequences. Events such as major vascular complications or acute kidney injury may directly affect survival through irreversible organ damage, functional decline, or delayed recovery. Conversely, the impact of new permanent pacemaker implantation remains more controversial and may depend on additional factors such as pacing dependency, ventricular pacing burden, and underlying conduction disease rather than device implantation itself.

The study has several notable strengths. Conducted within the framework of a randomized clinical trial with prospectively collected data and systematically adjudicated clinical events, it provides a robust assessment of outcomes in a contemporary TAVI population. Most importantly, by restricting the analysis to patients surviving the first 30 days, the authors were able to isolate the prognostic weight of early nonfatal complications from early mortality and thereby address an important limitation of previous studies. The inclusion of patient-reported quality-of-life assessment further expands the evaluation of procedural outcomes beyond survival. However, as a post-hoc analysis, the study remains exploratory and therefore cannot establish causal relationships. The relatively small number of individual complications limited the statistical power to reliably assess the prognostic contribution of each nonfatal event, highlighting the need for larger prospective studies. Finally, quality of life was assessed using the generic SF-12 rather than the disease-specific Kansas City Cardiomyopathy Questionnaire, potentially limiting sensitivity.

From a clinical perspective, early nonfatal complications represent more than transient procedural events, supporting their relevance as meaningful markers of long-term outcomes. As TAVI indications continue to expand, integrating these events into postprocedural risk stratification may help guide a more individualized follow-up strategy after hospital discharge. Whether targeted surveillance or specific interventions can mitigate this excess risk remains an important area for future investigation.

## Review Statement

Marisa Avvedimento, MD, is an associate deputy editor for *Structural Heart*. She had no role in the review of this manuscript.

## Disclosure Statement

The author has no conflicts to declare.
